# Erratum to “Evaluation of Immune System Components in Dogs With Protein‐Losing Enteropathy Compared to Healthy Controls”

**DOI:** 10.1111/jvim.70262

**Published:** 2025-10-09

E. Moore, K. Thelen Strong, and S. A. Jablonski, “Evaluation of Immune System Components in Dogs With Protein‐Losing Enteropathy Compared to Healthy Controls,” *Journal of Veterinary Internal Medicine* 39, no. 5 (2025): e70245, https://doi.org/10.1111/jvim.70245.

In the above‐mentioned article, Figure 1 (A–C) and Figure 3 (A–E) had labeling errors. There were no methodological errors. The correct figures are displayed here.

**Figure 1A. Boxplot showing serum concentrations of IgA in healthy control dogs and dogs with PLE. Median and interquartile range shown. HC: healthy control; PLE: protein‐losing enteropathy**.

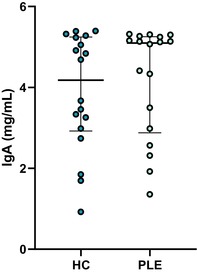

**Figure 1B. Boxplot showing serum concentrations of IgG in healthy control dogs and dogs with PLE. Median and interquartile range shown. HC: healthy control; PLE: protein‐losing enteropathy**.

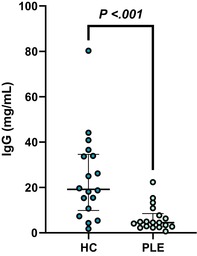

**Figure 1C. Boxplot showing serum concentrations of IgG in healthy control dogs and dogs with PLE. Median and interquartile range shown. HC: healthy control; PLE: protein losing enteropathy**.

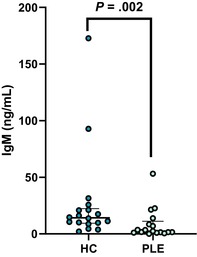

**Figure 3A. Boxplot depicting relative gene expression of CD3e in peripheral blood mononuclear cells of healthy control dogs and dogs with PLE. Median and interquartile range shown. HC: healthy control; PLE: protein‐losing enteropathy.**

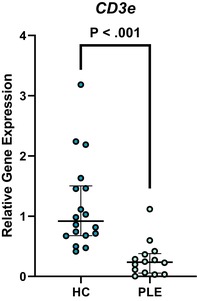

**Figure 3B. Boxplot depicting relative gene expression of CD4 in peripheral blood mononuclear cells of healthy control dogs and dogs with PLE. Median and interquartile range shown. HC: healthy control; PLE: protein‐losing enteropathy.**

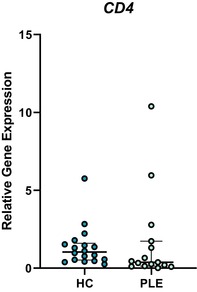

**Figure 3C. Boxplot depicting relative gene expression of CD5 in peripheral blood mononuclear cells of healthy control dogs and dogs with PLE. Median and interquartile range shown. HC: healthy control; PLE: protein‐losing enteropathy**.

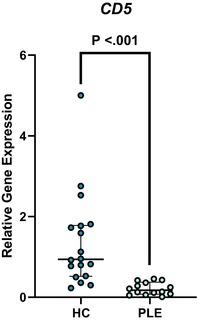

**Figure 3D. Boxplot depicting relative gene expression of CD8 in peripheral blood mononuclear cells of healthy control dogs and dogs with PLE. Median and interquartile range shown. HC: healthy control; PLE: protein‐losing enteropathy**.

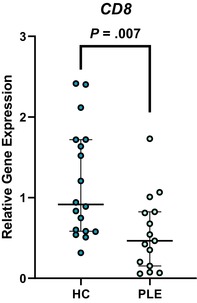

**Figure 3E. Boxplot depicting relative gene expression of CD21 in peripheral blood mononuclear cells of healthy control dogs and dogs with PLE. Median and interquartile range shown. HC: healthy control; PLE: protein‐losing enteropathy**.

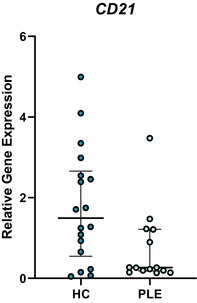

We apologize for the errors.

